# The H1047R point mutation in p110 alpha changes the morphology of human colon HCT116 cancer cells

**DOI:** 10.1038/cddiscovery.2015.44

**Published:** 2015-10-19

**Authors:** G Wan, C Pehlke, R Pepermans, JL Cannon, D Lidke, A Rajput

**Affiliations:** 1 Division of Surgical Oncology, Department of Surgery, University of New Mexico, Albuquerque, NM, USA; 2 Spatiotemporal Modeling Center, Comprehensive Cancer Center, University of New Mexico, Albuquerque, NM, USA; 3 Division of Molecular Medicine, Department of Internal Medicine, University of New Mexico, Albuquerque, NM, USA; 4 Department of Molecular Genetics Microbiology, University of New Mexico, Albuquerque, NM, USA; 5 Department of Pathology, University of New Mexico, Albuquerque, NM, USA

## Abstract

The class IA phosphatidylinositol 3-kinases (PI3K) is involved in controlling changes in cell morphology, which is a highly coordinated cellular event. This event is powered by actin filament polymerization and remodeling. The gain-of-function mutations in the catalytic subunit of p110*α* of class IA PI3K, which occur in up to one-third of human colorectal cancers (CRCs), are capable of causing dysregulation of cell signaling and thus may result in the alteration in cell morphology and motility and in turn cause cancer metastasis. *In vivo* studies have demonstrated that cell lines bearing the H1047R point mutation, the most frequent cancer-specific mutation in the kinase domain of p110*α*, are more metastatic than cells carrying wild-type p110*α*. In the current study, we show that the H1047R in p110*α* of PI3K decreases F-actin polymerization, increases the formation of filopodia and significantly changes the cell morphology in HCT116 cancer cells. The anti-apoptotic protein B-cell lymphoma 2 (Bcl-2), which is also involved in actin polymerization and cell migration, is downregulated by the H1047R mutation in p110*α*. Our data suggest that the H1047R mutation in PI3K is responsible for the rearrangement of the cytoskeleton, alteration in cell morphology and enhancing cell motility, and that Bcl-2 may be involved in the H1047R mutation-mediated morphological changes and increased migratory capability.

## Introduction

The dysregulation of the phosphoinositide 3-kinase (PI3K) signaling pathway has been implicated in the progression and metastasis of human cancers, including colorectal cancers (CRCs), and can frequently be induced by genetic mutations in class IA PI3K. In fact, gain-of-function mutations in PI3K have been found in nearly one-third of all human CRCs.^1–4^ Reports have shown that mutations in p110*α*, the catalytic subunit of PI3K, increase cell migration and the invasive capability of tumor cells.^5,6^ *In vivo* studies have demonstrated that cells bearing p110*α* mutations in PI3K were more metastatic than cells carrying wild-type (WT) PI3K in an orthotopic mouse model of colon cancer.^7^ Clinically, studies have shown a significant correlation between the mutations in *PIK3CA*, the p110*α*-encoding gene, and the survival of CRC patients. Patients carrying a *PIK3CA* mutation have a higher rate of disease relapse than patients lacking p110*α* mutations.^8^ Moreover, it has been reported that these mutations cause a gain of enzymatic fun,^3,4^ which in terms of cancer cell survival, may depend on the type of p110*α* mutations.^5,6^ These cancer-specific mutations in class IA PI3Ks are located in two specific ‘hotspot’ regions: in the helical domain or in the kinase domain of the p110*α* catalytic subunit. These ‘hotspot’ mutations have been identified in CRCs and account for 80% of p110*α*-bearing mutations.^2^ The most frequent mutation in the p110*α* kinase domain is at position 1047 where histidine is frequently substituted with arginine (H1047R).^1^

Many studies have demonstrated that PI3K is required for the remodeling of actin filaments induced by growth factors,^9,10^ Ras,^9,10^ G-protein-coupled receptors,^11^ integrins^12^ and insulin.^13,14^ It is one of the most important actin cytoskeleton regulators. Thus, any dysregulation involved in the PI3K pathway could affect cellular morphology and motility. Qian *et al.*^15^ have shown that activation of PI3K alone is sufficient to remodel actin filaments, which then increase cell migration through the activation of protein kinase B (PKB) or serine/threonine-specific protein kinase (Akt) and p70S6K1 in chicken embryo fibroblast (CEF) cells.

Although these findings have shown that the mutations in p110*α* of PI3K increase cell migration and tumor metastasis, the mechanisms behind these actions are still unclear. Furthermore, there is no direct evidence showing that PI3K mutations are involved in actin cytoskeleton reorganization. In this study, we focused on the relationship between the H1047R point mutation in the p110*α* kinase domain of PI3K and cell morphology. Our experiments were designed to determine whether the H1047R mutation is capable of: (1) changing the cell morphology of HCT116 cells and (2) reorganizing the actin cytoskeleton, which may explain why CRC cells harboring the H1047R mutation are more metastatic than WT cells. Our results indicate that the H1047R mutation in PI3K decreases F-actin polymerization, while significantly increasing cellular filopodia formation and cell motility, as compared with WT PI3K. Further experiments were designed to investigate what cytoskeletal regulatory factors are involved in the H1047R mutation-mediated cell morphological changes. Our data suggest that B-cell lymphoma 2 (Bcl-2) may be involved in the H1047R mutation-mediated cell morphological changes and increased cell migration.

## Results

### The H1047R mutation in p110*α* changes the cell morphology and the appearance of actin filaments in HCT116 cells

**Figure 1:**
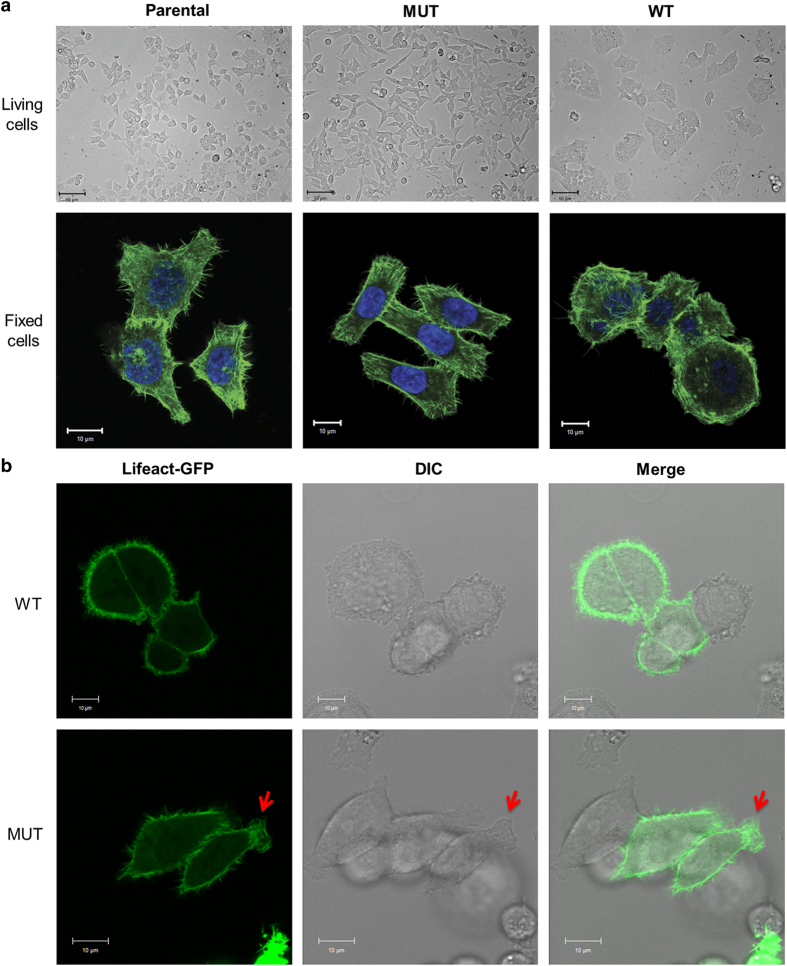
Cell morphology of HCT116 cells is altered by the H1047R mutation in the p110*α* kinase domain of PI3K. (**a**) Cell morphology of HCT116 cells. Top panel: cell morphologies of live parental, WT and MUT HCT116 cells captured at a ×20 magnification. Bottom panel: confocal images parental, WT and MUT HCT116 cells captured at a ×63 magnification. Cells were fixed and stained for F-actin (green). Nuclei were stained with DAPI (blue). (**b**) Movement and morphology of live HCT116 WT (top) and MUT (bottom) cell at a ×40 magnification. Cells were transfected with Lifeact-GFP and cultured in a chambered cover glass dish for 24 h, and fluorescence and DIC images were acquired over 20 min.

The polymerization and organization of actin microfilaments, the major structural filament of cytoskeleton in cells, determine the overall shape of the cell,^16^ contribute to its internal organization and have a key role in the morphological change of cells.^17^ For certain cell types, this morphological change is indispensable to gain the proper function in the tissue.^18,19^ In other words, the changes in the actin cytoskeleton structure could result in dysregulated function, for example, increasing tumor cell migration. To investigate the effect of the H1047R mutation on cell morphology and actin cytoskeleton structure, we used cell lines harboring either WT or mutant (MUT; H1047R) p110*α* of PI3K, which were generated by asymmetric deletion of the *PIK3CA* allele from the CRC parental cell line HCT116. The cells were stained for F-actin with Alexa Fluor 488 Phalloidin and the cell morphology was determined by imaging. The morphology of HCT116 MUT cells was considerably different than that of WT cells (Figure 1). Unlike WT cells, which normally exhibit a round and more clumped morphology, MUT cells became elongated and actin filaments appeared to align along the length of the cell, adopting a more fibroblastic and less clumped morphology.

Cell protrusion involves the extension of cellular membranes. Its physical backbone is constituted by actin filaments. It usually occurs in response to the stimulation of chemoattractive signals in the microenvironment. However, some cells extend protrusions in a probing, exploratory manner in the absence of directional stimulation.^20^ To understand whether the H1047R mutation could also affect the behavior of cell protrusions in the absence of directional stimulation, we tracked the motion of living HCT116 WT and MUT cells expressing Lifeact-green fluorescent protein (GFP)^21^ under high magnification (Figure 1b and Supplementary Movies 1 and 2). The WT cells exhibited a rounded shape with no obvious morphological polarity and their protrusions seemed to be random. On the other hand, however, HCT116 MUT cells displayed an elongated shape with stronger protrusion points primarily at two end sides of the cells (Figure 1b; red arrows).

### The H1047R mutation in p110*α* induces a change in cell projections and increases the formation of actin filopodia

**Figure 2:**
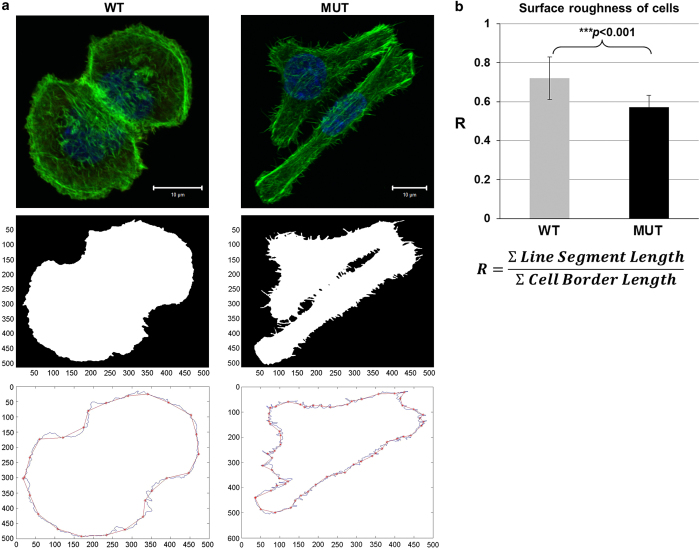
The H1047R mutation in the p110*α* kinase domain of PI3K increases the number of filopodia-like actin filaments. (**a**) Quantification of filopodia-like actin filaments in HCT116 WT and MUT cells. Original confocal images of F-actin-stained (green) HCT116 WT (top panel, left) and MUT cells (top panel, right), cell masks created by Matlab (middle panel), and Matlab-drew cell outline or border line (blue) as well as cell straight line segment (red; bottom panel), which are uniform 5-*μ*m length and were superimposed along the cell border line. The cell roughness (*R*) was used as a surrogate measure for the estimated density or amount of filopodia around the cell borders, which are the ratios between the sum of the lengths of the line segments and the total cell border length (**b**), the summary of cell surface roughness (*R*). Over 40 cells of each cell line were used for the analysis. Statistical analysis was performed using a two-tail Student's *t*-test, where *P*=0.001.

Cell projections are dynamic and morphologically varied extensions of the plasma membrane, supported by the actin cytoskeleton, which are essential for cell migration. The higher-order actin-based structures are lamellipodia and filopodia. Filopodia are thin membrane protrusions that are oriented with the barbed end of their actin filaments towards the protruding membrane and extend beyond the leading edge of lamellipodia in migrating cells.^22^ Our image analysis data showed that WT and MUT HCT116 cells display different cell projections (Figure 2). To quantify the filopodia projections, we generated a Matlab script as described in a previous report.^23^ By comparing the value of *R*, which measures the surface roughness of cells, we found that MUT cells had lower *R*-values (0.56 on average), whereas *R*-values of WT cells were closer to 1 (0.91 on average; Figure 2a). The evaluation of the statistical significance on the numbers of filopodia protrusions around the cell surface of WT and MUT cells, were significant (*P*<0.001) (Figure 2b). This difference corresponds to our finding that MUT cells have much more and longer filopodia, which are protruding out of the cell membrane, compared with WT cells.

### The H1047R mutation in p110*α* decreases actin polymerization and increases cell migratory capability

**Figure 3:**
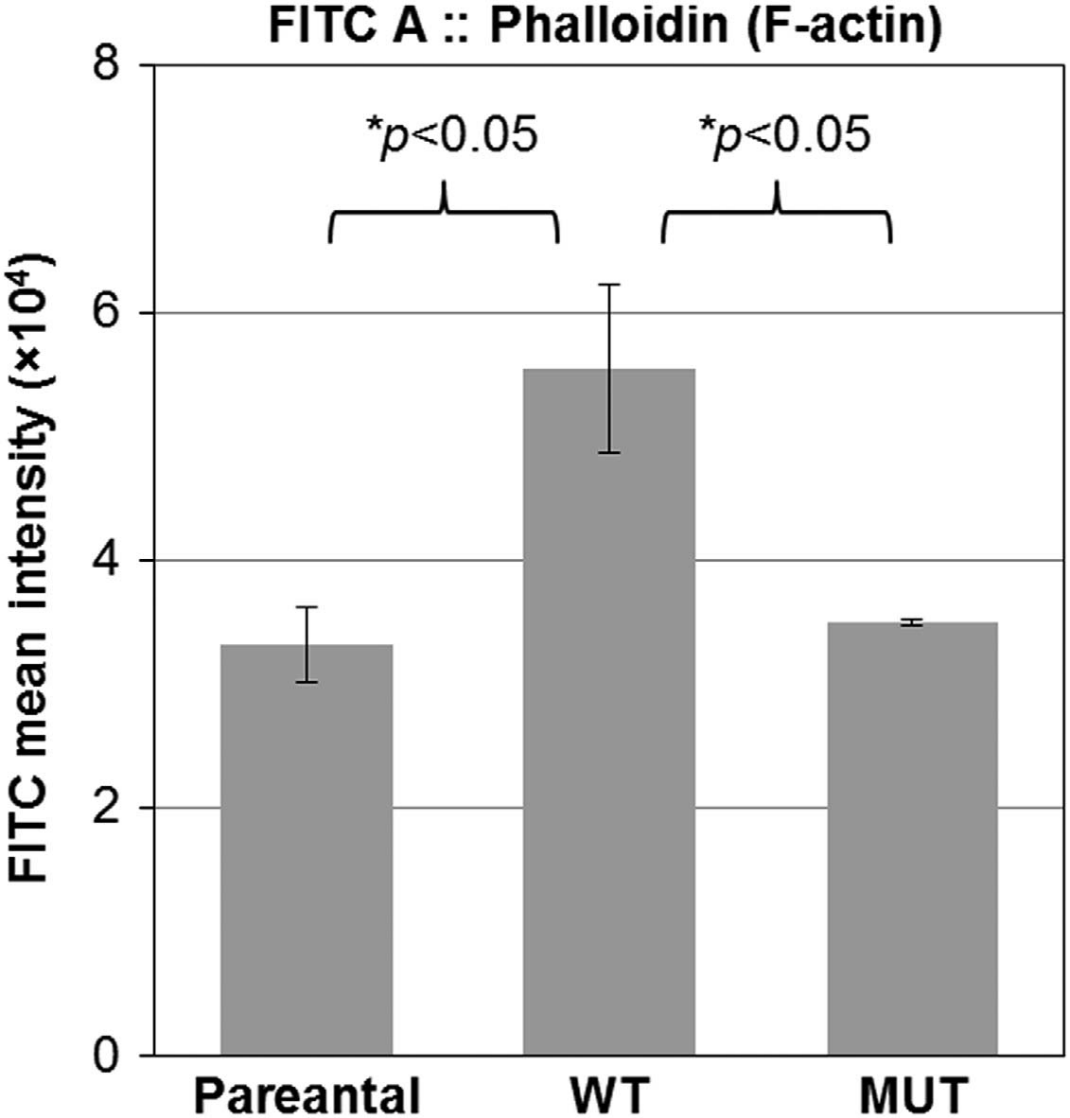
The H1047R mutation in the p110*α* kinase domain of PI3K decreases the actin polymerization. Cells were stained with Alexa Flour 488-conjugated phalloidin and their mean FITC::Phalloidin intensities were measured by flow cytometry. The mean intensities of FITC (Alexa Flour 488)::Phalloidin indicate F-actin contents. The experiment was repeated three times, and each experiment included at least two duplications. This indicates that F-actin content in WT cells was significantly higher than that in parental or MUT cells (two-tail Student's *t*-test, *P*<0.05).

**Figure 4:**
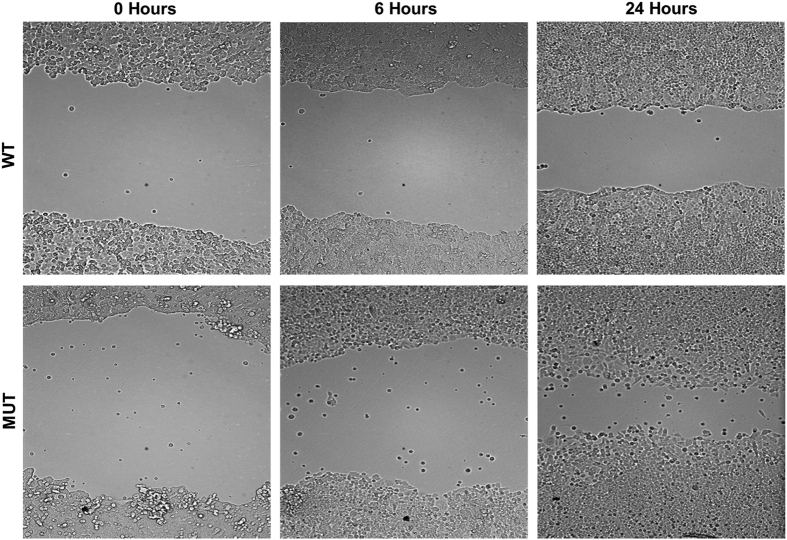
The H1047R mutation in the p110*α* kinase domain of PI3K increases cell migration. The migratory capability of HCT116 WT and MUT cells was measured by wound-healing assay. HCT116 MUT cells revealed the faster wound closure compared with WT cells at 24 h.

F-actin is a critical player in many cellular functions, ranging from cell motility and the maintenance of cell shape and polarity to the regulation of transcription. It is a linear actin polymer and is formed by polymerization of monomeric G-actin and the dynamics of actin polymerization and depolymerization is critical for cell migration.^16^ To test whether the H1047R mutation in p110*α* affects the levels of actin polymerization, F-actin levels were measured in HCT116 parental, WT and MUT cells by flow cytometry (Figure 3). The results indicated that the level of F-actin in parental and MUT cells was significantly decreased as compared with that in WT cells. Moreover, MUT cells were found to have increased motility as compared with WT cells when assayed in a wound-healing experiment (Figure 4).

### Activation of PI3K by H1047R mutation in p110*α* contributes to changes in cell morphology

It has been reported that the H1047R mutation in p110*α* will result in constitutive activation of PI3K.^2^ Activation of PI3K directly regulates downstream signal transduction through excessive production of phosphatidylinositol (3,4,5)-trisphosphate (PtdIns(3,4,5)*P*_3_) (PIP_3_). The important second messenger PIP_3_, is virtually undetectable in quiescent cells and is only transiently produced in the plasma membrane of stimulated cells by the class-1 PI3Ks.^24^ Thus, the increased PI3K kinase activity by mutated p110*α* will result in an overabundance of PIP_3_ in cells.^1,4,8^ In order to compare PI3K activity in HCT116 WT and MUT cells, we measured their intracellular levels of PIP_3_ and phosphorylation levels of Akt, one of the most important downstream substrates of PI3K signaling pathway. The MUT cells showed both higher levels of PIP_3_ (Supplementary Figure 1) and phosphorylation of Akt (Supplementary Figure 2) as compared with WT cells.

The PI3K signaling pathway can be negatively regulated by the tumor suppressor PTEN (phosphatase and tensin homolog). PTEN converts PIP_3_ in the cytoplasm to PIP_2_, thereby directly antagonizing the activity of PI3K.^25^ Inactivation of PTEN also results in constitutive activation of the PI3K pathway and a subsequent increase in protein synthesis, cell cycle progression, migration and survival.^25,26^ Thus, the PTEN status is important for the experimental setting, it has to be normal and the expression level was required to be equable in these isogenic cell lines of HCT116 cells. Our results showed no difference in the level of PTEN in parental, WT and MUT HCT116 cells (Supplementary Figure 2) and that HCT116 cell line possesses WT PTEN (data not show).

In addition, it is known that p85, the regulatory subunit of PI3K, has an important role in stabilizing the catalytic subunit p110.^27^ Therefore, we also measured the expression levels of p110*α* and p85*α* in all the cell lines and found similar expression level of p110*α* in all three cell lines, but higher expression level of p85*α* in parental and MUT cells than in WT cells (Supplementary Figure 2).

**Figure 5:**
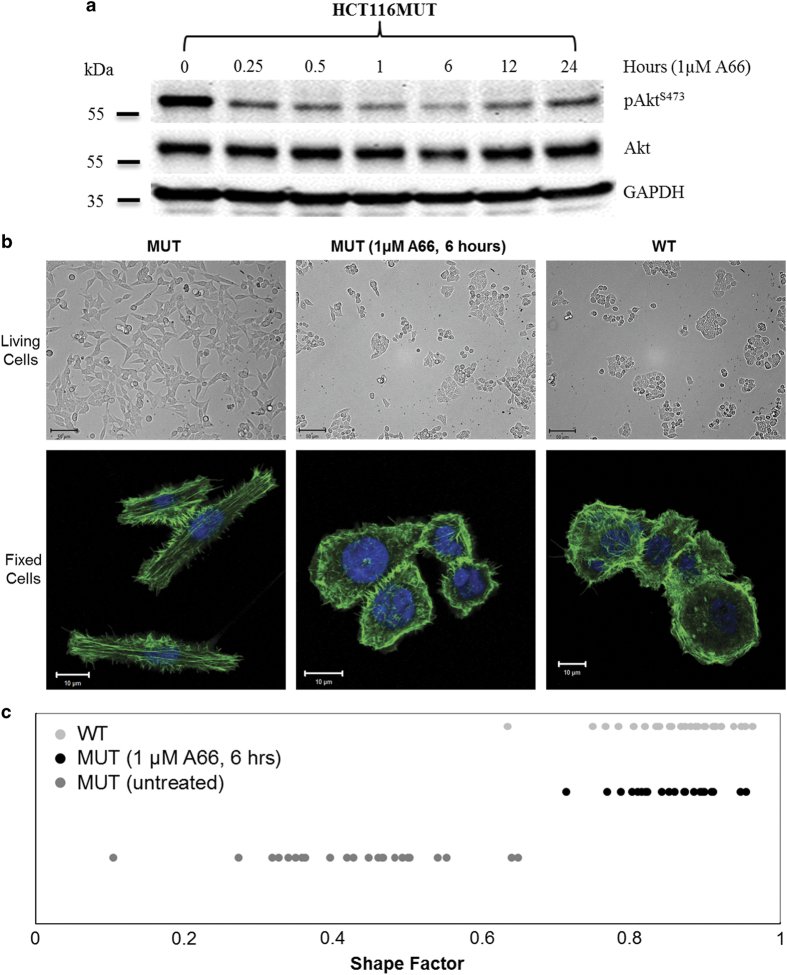
Cell morphology of HCT116 MUT cells resemble that of WT cells following inhibition of PI3K-p110*α*. (**a**) Time-dependent inhibition of A66 on PI3K-p110*α*. HCT116 MUT cells were cultured in 1 *μ*M A66 containing medium for the indicated time points and immunoblotting analysis was used to determine the phosphorylation of Akt. The maximal inhibition effect was at 6 h. (**b**) Cellular morphology of HCT116 MUT cells in the presence or absence of A66. HCT116 MUT cells were cultured on coverslips and incubated for 6 h in the medium presence or absence of 1 *μ*M A66. Top panel: cell morphology of the living MUT (±A66) and WT HCT116 cells (×20 magnificence). Bottom panel: confocal images of fixed MUT (±A66) and WT HCT116 cells (×63 magnificence). For the confocal images, cells were fixed and stained for F-actin (green) and nuclei (blue). (**c**) Shape factor of HCT116 MUT cells in the presence or absence of A66. Quantitative analysis of the cell shape of MUT cells, treated with/without A66, was conducted using shape factors. Shape factors were determined by the area, perimeter and circularity of individual cells. Untreated WT cells were used as the control. For each sample, at least 20 individual cells were included in this assay.

Next, we investigated whether the H1047R mutation-mediated gain of function of PI3K is directly involved in the morphologic change of MUT cells. To do this, A66 was utilized because it exhibits greater specificity in inhibiting p110*α* as compared with other p110*α* inhibitors. Thus, other class-1 PI3Ks, class-II PI3Ks, class-III PI3K and PI4Ks (phosphoinositide 4-kinases) are unaffected and maintain their function in growth factor signaling.^28^ MUT cells were either treated or not treated with 1*μ*M of A66 at several time points (0, 0.25, 0.5, 1, 6, 12 and 24 h). Inhibition of A66 on kinase activity was confirmed by the immunoblotting analysis of the phosphorylation level of Akt, with maximum inhibition reached around 6 h (Figure 5a). HCT116 MUT, treated for 6 h with the inhibitor, adapted a cellular morphology similar to that of WT cells (Figure 5b). We use the shape factor to evaluate the overall morphologic alteration of HCT116 MUT cells at 6 h inhibition of A66. Shape factors were determined and are summarized in Figure 5c. Untreated cells had an average shape factor value of 0.43, and displayed an elongated morphology. In contrast, cells treated for 6 h with 1.0 *μ*M of A66 had a larger shape factor (average 0.86), and had the morphology similar to that of WT cells (average shape factor: 0.86).

### The H1047R mutation in p110*α* decreases Bcl-2 expression, which may contribute to reduced actin polymerization and remodeling of cytoskeleton

**Figure 6:**
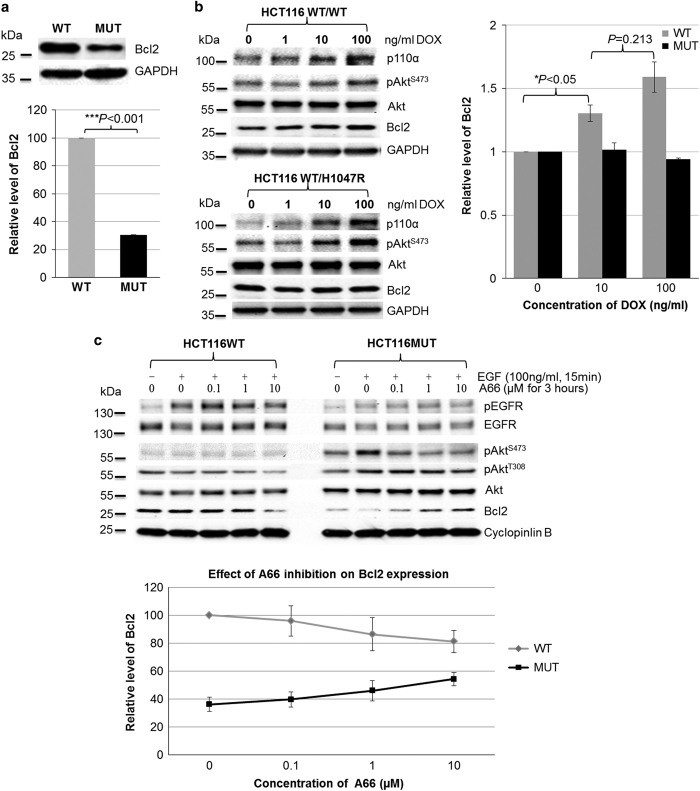
The H1047R mutation in the p110*α* kinase domain of PI3K affected Bcl-2 expression level. (**a**) Endogenous level of Bcl-2 in HCT116 WT and MUT cells. Endogenous level of Bcl-2 in HCT116 WT and MUT cells was measured by immunoblotting analysis (top). The graph shows the quantification of Bcl-2 bands normalized by glyceraldehyde 3-phosphate dehydrogenase (GAPDH) for three individual experiments (bottom). The Bcl-2 level in HCT116 WT cells was at least three times higher than in HCT116 MUT cells. (**b**) Effects of overexpressing WT-p110*α* and H1047R-p110*α* on Bcl-2 levels. HCT116 WT cells, which were transfected with the Tet-On 3G-inducible plasmids pTRE3G-BI-mCherry/p110*α*^WT^ or pTRE3G-BI-mCherry/p110*α*^H1047R^, were cultured in the medium containing indicated concentration of doxycycline (DOX; left). Their Bcl-2 levels were measured by immunoblotting analysis (left). The graph shows the quantifications of Bcl-2 bands of two different clones from the individual cell lines (right). Overexpression of WT-p110*α* resulted in a DOX dose-dependent increase in Bcl-2 levels, however, Bcl-2 levels were not verified by overexpression of H1047R-p110*α*. (**c**) Effect of p110*α* inhibition on Bcl-2 levels. HCT116 WT and MUT cells were serum starved overnight, subsequently cultured in the presence of A66 at the indicated concentrations for 3 h, and then followed by 100 ng/ml EGF stimulation for 20 min. Immunoblotting analysis was used to measure Bcl-2 levels in HCT116 WT (left) and MUT (right) cells. The graph shows the quantifications of expression Bcl-2 levels in HCT116 WT and MUT cells on treatment of A66 (bottom panel). Data are the average of two independent experiments. Bcl-2 levels in HCT116 MUT cells was A66 dose-dependently increased, however, an A66 dose-dependent decrease was observed in HCT WT cells.

It is unclear how the single H1047R amino-acid substitution in the kinase domain of p110*α* can markedly decrease actin polymerization in HCT116 cells, resulting in a change in cell morphology. Recently, Ke *et al.*^29^ found that Bcl-2 inhibits cell adhesion, spreading and motility by enhancing actin polymerization. Therefore, it was very interesting whether the H1047R mutation in p110*α* can affect the level of Bcl-2 in HCT116 cells. We compared the endogenous level of Bcl-2 in HCT116 WT and MUT cells by immunoblotting analysis. We found that cells bearing WT-p110*α* possess a higher expression level of Bcl-2 as compared with that in MUT cells (Figure 6a). Next, we tested whether and how the Bcl-2 levels in HCT116 WT cells can be altered by the increased expression of WT- or H1047R-p110*α*. HCT116 WT cells transfected with Tet-On 3G-inducible plasmids, pTRE3G-BI-mCherry/p110*α*^*WT*^or pTRE3G-BI-mCherry/p110*α*^*H1047R*^, were cultured for 24 h in the medium containing 0, 10 and 100 ng/ml of doxycycline (DOX), respectively. The levels of Bcl-2 were measured by immunoblotting analysis and quantified using Imaging Lab Software (Bio-Rad laboratories, Hercules, CA, USA). Figure 6b shows that Bcl-2 expression was increased following the induced expression of WT-p110*α*, whereas the overexpression of H1047R-p110*α* had no effect. Furthermore, we examined whether the inhibition of p110*α* alters the expression of Bcl-2. HCT116 MUT cells showed an A66-dose-dependent increase in Bcl-2 expression, however, WT cells showed an A66-dose-dependent decrease in Bcl-2 expression (Figure 6c).

**Figure 7:**
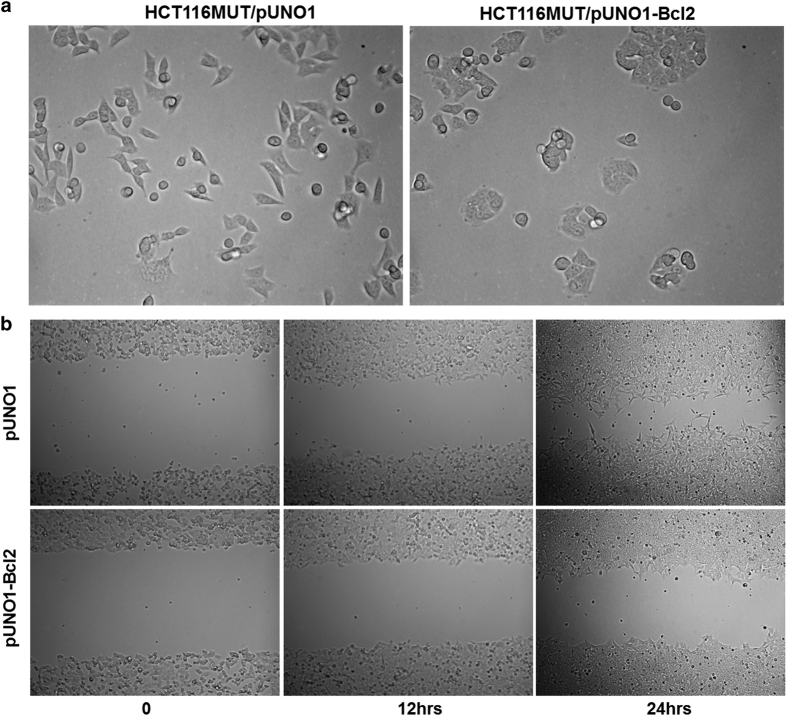
Overexpression of Bcl-2 altered cell morphology and delayed cell migration of HCT116 MUT cells. (**a**) Overexpression of Bcl-2 changed cell morphology of HCT116 MUT cells. HCT116 MUT cells were stable transfected with the pUNO1 or pUNO1-Bcl-2 plasmid and imaged at ×20 magnification. Cell morphology became rounded and cells aggregated together when Bcl-2 was overexpressed. (**b**) Cell migration of HCT116 MUT cells was inhibited by Bcl-2 overexpression. HCT116 MUT cells were stably transfected with the pUNO1 or pUNO1-Bcl-2 plasmid. Wound-healing assay was performed as described in the Materials and methods. Overexpression of Bcl-2 delayed the wound closure at 12 and 24 h in the stable transfected HCT116 MUT cells as compared with the vector transfected HCT116 MUT cells.

In order to determine whether Bcl-2 is directly involved in the H1047R-p110*α*-mediated cell morphological changes, HCT116 MUT cells were transfected with pUNO1 or pUNO1-Bcl-2 plasmids. Overexpression of Bcl-2 resulted in HCT116 MUT cells adapting a more circular shape and caused them to clump together (Figure 7a). Moreover, overexpression of Bcl-2 delayed the wound closure in HCT116 MUT cells (Figure 7b).

These data thus suggest that the H1047R mutation in p110*α* of PI3K decreases Bcl-2 expression levels and that the downregulation of Bcl-2 is involved in the H1047R-p110*α*-mediated cell morphological changes and increased migratory capability of HCT116 cells.

## Discussion

Recent studies have demonstrated that the gain of function induced by mutations in PI3K not only results in enhanced migration and invasion of cancer cells, but also metastasis of cancer *in vivo*.^4,5,6^ Guo *et al.*^7^ have shown that *in vivo*, CRC cells bearing the H1047R mutation in p110*α* of PI3K have a higher rate of metastasis to the liver than cells bearing WT PI3K. Our current study has shown that the H1047R mutation in p110*α* of PI3K causes CRC HCT116 cells to become more motile compared with the WT cells. This increased migration may contribute to metastatic capability. We have also confirmed that one of these important gain-of-function mutations, H1047R, in the catalytic subunit p110*α* of PI3K, increases the production of PIP_3_ and the phosphorylation of Akt. These results are consistent with findings in previous reports.^6,7^ Importantly, our results show that the H1047R mutation decreases F-actin polymerization, increases filopodia formation and results in a marked change in cell morphology. This morphological change, however, was abrogated by the inhibition of p110*α*. The change in cell morphology, mediated by the H1047R mutation in p110*α*, may grant HCT116 MUT cells their increased ability to migrate. This is, to the best of our knowledge, the first demonstration that the H1047R kinase mutation in 110*α* of PI3K is related to the reorganization of the actin cytoskeleton and the resulting changes in cell morphology, which possibly contributes to the enhanced migratory capacity. Furthermore, we have found that downregulation of Bcl-2 can be caused by the H1047R mutation in p110*α* and that this downregulation may be involved in the H1047R mutation-induced alteration of actin cytoskeleton and cell motility.

Cell migration is the key cellular feature of cancer metastasis. It is a highly coordinated cellular event that is powered by actin polymerization and remodeling of actin filaments.^16,30^ Studies have suggested that higher levels of actin polymerization result in lower cell motility.^15,31^ We have shown that F-actin levels were decreased in HCT116 parental and MUT cells compared with WT cells (Figure 3). This could explain why the H1047R mutation in p110*α* of PI3K can increase cell motility.

It is known that in order for cells to change their morphology or acquire a migratory phenotype, they must undergo extensive actin cytoskeleton remodeling, which is regulated by over 100 actin-binding proteins and controlled by many cellular signaling transduction mediators. PI3Ks and Bcl-2 are two important players in regulating cell survival through two distinct cell signaling pathways.^32–34^ However, both of them have also been reported to be involved in cell adhesion, cytoskeletal rearrangement and cell motility.^29–34^ These functions can be dissociated from their survival or anti-apoptotic function. Ke *et al*.^29^ reported that Bcl-2 enhances actin polymerization and proposed a mechanism for the enhanced actin polymerization by Bcl-2: Bcl-2 expression may regulate the gelsolin-mediated activity of F-actin severing.^31^ Interestingly, it was reported that overexpression of Bcl-2 results in cells acquiring a rounded shape and increased actin expression in the prostate cancer cell line PC12.^35^ This agrees with our finding that WT-p110*α* HCT116 cells possess high levels of Bcl-2 and display more circular cell morphology compared with MUT cells. Moreover, there is data demonstrating that inhibition of PI3K result in a 45% decreases in Bcl-2 promoter activity, and the activation of Bcl-2 promoter is controlled by Akt/cAMP-response element-binding activity.^36^ In other words, intracellular Bcl-2 expression could potentially be altered by either inhibition or dysfunction of PI3K. Our data suggest that H1047R mutation in p110*α* mediates the downregulation of Bcl-2 (Figure 6a). Furthermore, several reports have shown that loss of Bcl-2 expression correlates with tumor recurrence in CRC.^37^ A high level of Bcl-2 is also predictive of relapse-free survival of stage II CRC.^38^ These findings suggest that the H1047R mutation enhanced liver metastasis of CRC^7^ may be related to the low level of Bcl-2 expression. In addition, our results show that Bcl-2 levels were upregulated in HCT116 WT cells upon overexpression of WT-p110*α* in DOX dose-dependent manner. However, overexpression of H1047R-p110*α* had no effect on Bcl-2 levels, although DOX dose-dependent overexpression of H1047R-p110*α* resulted in hyperactivity of PI3K (higher phosphorylation level of Akt; Figure 6b). On the other hand, cellular Bcl-2 levels of HCT116 WT cells were decreased through A66-mediated inhibition of p110*α*, whereas A66 treatment of HCT116 MUT cells resulted in a dose-dependent increase in Bcl-2 levels (Figure 6c). Interestingly, phosphorylation of Akt was enhanced by EGF stimulation in cells bearing the H1047R mutation in p110*α*, whereas the activation of Akt, in HCT116 WT cells, was not EGF dependent. These observations suggest that WT-p110*α* and H1047R-p110*α* may regulate Bcl-2 expression by different signaling pathways or mechanisms which need to be further investigated.

The rounded and clumped cell morphology of HCT116 MUT cells was induced by overexpression of Bcl-2 (Figure 7a). This suggests that downregulation of Bcl-2, in HCT116 MUT cells, probably has an important role in the H1047R mutation-mediated reorganization of the actin cytoskeleton and cell morphologic changes. It has been reported that knockdown of anti-apoptotic Bcl-2 proteins directly inhibits the migration and invasion of the CRC cells HT29 and SW480, independent of their cell death induction or effects on proliferation.^39^ However, our results show that overexpression of Bcl-2 in HCT116 MUT cells impairs gap closure in a wound-healing assay (Figure 7b). It is known that SW480 cell line expresses WT PI3K and that HT29 cells bear the P449T^b^ mutation in p110*α*.^40^ All of these observations further indicate the multiple and complex functions of Bcl-2 and PI3K, as well as that different phenotypes of p110*α* may regulate actin cytoskeleton and cell migration by cooperating with Bcl-2 through different mechanisms.

It is hard to ignore the function of secondary messenger PIP_3_. Janetopoulos *et al.*^41^ demonstrated that the regulation of PIP_3_ has a central role in changing the cell morphology during cytokinesis. It has also been reported that PIP_3_ has an essential role in many actin-based cellular processes, encompassing cell migration and invasion.^42,43^ Moreover, a recent report demonstrated that the overexpression of a constitutively active form of PI3K, v-P3K, is sufficient to induce the remodeling of actin filaments to form lamellipodia and filopodia in CEF cells.^15^ In the present study, our results clearly confirm that the activation of PI3K and intracellular accumulation of PIP_3_ by H1047R mutation in p110*α* not only results in elongated cell shape but also significantly changes the actin cytoskeleton structure (Figures 1 and 2). In other words, the H1047R mutation results in the cells gaining many more filopodia filaments. PIP_3_ has been shown to accumulate at the tips of filopodia in dendritic cells.^44^ This induced formation of filopodia was reported to be dependent on PIP_3_ binding via the PH-domain of myosin-X, which is primarily found at the tips of filopodia.^45^ Filopodia have important roles in sensing extracellular signals, migration and cell–cell interactions.^22^ Moreover, an increased density of filopodia has been described in cancer.^46^ Recent research has shown that filopodia-associated genes are upregulated in breast carcinomas with a poor prognosis,^47^ and that filopodia stimulate cell migration in many cell types.^48^

**Figure 8:**
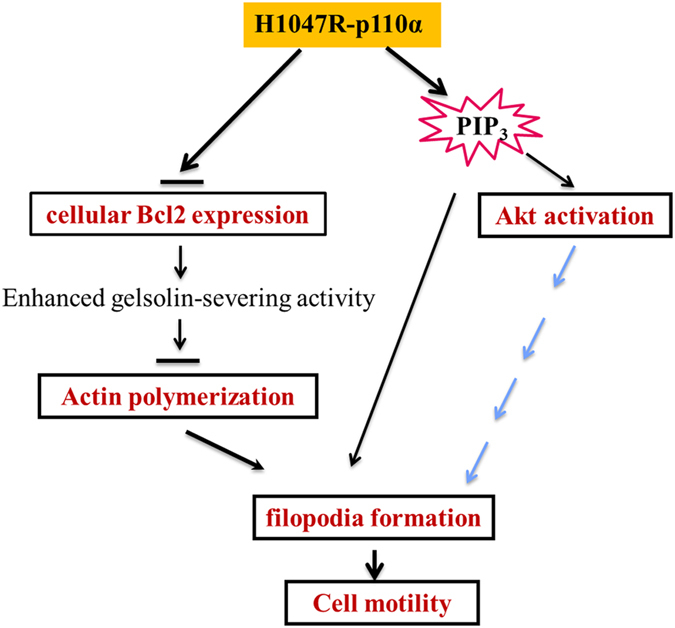
Model for H1047R-p110*α*-induced morphological change and increased cell motility in HCT116 cells. The H1047R mutation in p110*α* causes the downregulation of Bcl-2, which results an enhanced gelsolin-severing activity. This blocks actin polymerization, induces reorganization of actin cytoskeleton and increase filopodia formation. Moreover, the accumulation of PIP_3_ and hyperactivity of Akt may modify the structure of the cytoskeleton by the different mechanisms.

In conclusion, our findings indicate that the H1047R mutation reorganizes actin structure and in turn results in change of cell morphology. This function possibly contributes to the enhanced migratory capacity of HCT116 MUT cells. Although the underlying mechanism behind the morphological changes is complex, the H1047R-p110*α*-mediated cell morphological change and enhanced motility in HCT116 cells may be partially caused by the downregulation of the oncogene Bcl-2; and the cellular accumulation of PIP_3_ may be another import cause by different signaling pathway (Figure 8). Further studies are needed to investigate how the H1047R mutation in p110*α* causes the downregulation of Bcl-2 and to understand the role of Bcl-2 and PIP_3_ in the H1047R mutation-mediated CRC metastasis. Our results further confirmed that the accumulation of PIP_3_, Bcl-2 level and change in the appearance of cytoskeleton of cells are important aspects in regulating cell motility and, subsequently, metastasis. Thus, the PI3K pathway remains a desirable target for CRC therapeutics. Our results have implications for ongoing studies using therapeutics targeting both the PI3K/Akt and Bcl-2 pathways.

## Materials and methods

### Cell lines and culture medium

The HCT116 WT and HCT116 MUT CRC cell lines engineered to contain either the wild type (WT) or the H1047R mutant (MUT) *PIK3CA* allele, respectively, were gifts from Dr. Vogelstein and Dr. Velculescu (The Johns Hopkins Kimmel Cancer Center, Baltimore, MD). Both cell lines were generated from the parental cell line HCT116, which possesses both a MUT and WT *PIK3CA* allele, by targeted deletion of either the MUT or WT *PIK3CA* locus.^6^ The cells were cultured and maintained in McCoy’s 5A medium (Sigma, St. Louis, MO, USA) supplemented with 10% (v/v) fetal bovine serum at 37 °C in a humidified atmosphere of 95% air and 5% CO_2_.

### Plasmid constructs

To construct Tet-on 3G bidirectional-inducible expression plasmids of either WT-p110*α* or H1047R-p110*α*, we first amplified the full-length cDNAs (*PIK3CA*^*WT*^ or *PIK3CA*^*A3140G*^) encoding WT-p110*α* or H1047R-p110*α* from total RNA, which were extracted from HCT116 WT and MUT cell, respectively, using the One Step Reverse Transcriptional–PCR Kit (Cat. 210210, QIAGEN, Valencia, CA, USA). The following primers were used for the PCR amplification: forward, 5′- accggggcccagatctATGCCTCCACGACCATCATCAGG-3′ and reverse, 5′-gcggatcgatggatccTCAGTTCAATGCATGCTG-3′. The cDNAs (*PIK3CA*^*WT*^ or *PIK3CA*^*A3140G*^) were subsequently cloned into the pTRE3G-bi-mCherry vector using the In-Fusion HD Cloning Kit (PT5162, Clontech, Mountain View, CA, USA). The pCMV-Tet3G, encoding Tet-On 3G transactivator protein, was purchased from Clontech (Cat. 631335).

The pUNO1-hBcl-2 alpha (Cat. Puno1-hbcl2) and the empty pUNO1 vector (Cat. Puno1-mcs) were purchased from InvivoGen (San Diego, CA, USA).

### Transfection of LifeAct-GFP, pTRE3G-BI-mCherry/p110*α*^WT^, pTRE3G-BI-mCherry/p110*α*^H1047R^, pUNO1 and pUNO1-hBcl2alpha

Lifeact-GFP, as described by Riedl *et al*,^21^ is a versatile maker comprising a GFP-tagged F-actin-binding domain and can thus be used to visualize actin cytoskeleton in living cells. HCT116 WT and MUT cells were transfected with the Lifeact-GFP-encoding plasmid using the Lipofectamin 2000 transfection reagent (Life Technologies, Cat: 11668-019, Grand Island, NY, USA).

HCT116 WT cells, which have been previously transfected with pCMV-Tet3G, were transfected with pTRE3G-BI-mCherry/p110*α*^*WT*^ or pTRE3G-BI-mCherry/p110*α*^*H1047R*^ using the Xfect transfection reagent (Cat. 631317, Clontech).

HCT116 MUT cells were transfected with pUNO1 and pUNO1-hBcl2-alpha using the Xfect transfection reagent (Cat. 631317, Clontech).

### F-actin staining

For F-actin labeling, HCT116 WT and MUT cells were grown on coverslips for 24 h. Once at the desired confluence (50–60%), cells were fixed in 4% paraformaldehyde (PFA) in PBS for 15 min at room temperature. Following three washes with PBS, cells were permeablized in 0.1% Triton X-100 in PBS at room temperature for 10 min and subsequently washed three more times with PBS. Next, cells were incubated with Image-iT FX signal enhancer (I36933, Molecular Probe, Carlsbad, CA, USA) for 20–30 min to reduce nonspecific background staining by fluorescently conjugated antibodies or reagents. Following three washes with PBS, cells were incubated in 100–200 *μ*l of 1 : 80 PBS-diluted Alexa Fluor 488 phalloidin (A12379, Invitrogen, Grand Island, NY, USA) for 20 min in a light-tight covered 6-well plate. Next, the cells were rinsed in PBS and mounted onto glass coverslips using ProLong Gold reagent containing DAPI (P36935, Invitrogen). All steps are at room temperature. The coverslips were stored in the dark at 2–6 °C until they were imaged.

### Imaging

To characterize the overall cell morphology of living HCT116 parental, WT and MUT cells in the presence or absence of p110*α* inhibitor, individual images of living cells were captured randomly and in a blinded manner, on a Nikon TE2000 inverted microscope using a ×20 dry objective (Melville, NY, USA). Confocal images of HCT116 Parental, WT and MUT cells, which were fixed and stained for F-actin, were acquired using a Zeiss LSM510 META/Axio Observer confocal microscope (Carl Zeiss, Microimaging, Inc., Thornwood, NY, USA), with a 63×1.4 NA oil objective. Time-lapse images of live HCT116 WT and MUT cells, transfected with Lifeact-GFP, were acquired with a Zeiss LSM510/Axiovert 200 M confocal microscope using a ×40 W1.1 NA objective.

### Filopodia analysis

The degree of difference between the structures of filopodia in HCT116 WT and MUT cells was assessed using a custom Matlab (R2012b, The Mathworks, Natick, MA, USA) script. The analysis was similar to that performed by Mege *et al*.^23^ Straight line segments of uniform 5-*μ*m length were superimposed along the cell borders. The actual length of the cell border between the start and end points of these line segments was then measured. The ratio between the sum of the lengths of the 5 *μ*m line segments and the total cell border length, *R*, is a measure of the surface roughness of the cell, which corresponds to the number of filopodia present:

$$R=\frac{\text{Σ}\;\mathrm{Line}\;\mathrm{segment}\;\mathrm{length}}{\text{Σ}\;\mathrm{Cell}\;\mathrm{border}\;\mathrm{length}}$$

Therefore, an *R*-value close to 1 indicates a smooth surface, whereas an *R*-value close to 0 corresponds to a rougher surface.

### Immunoblotting analysis

Total protein concentrations of cell lysates were measured using the Protein Assay kit (Bio-Rad). A total of 20 *μ*g of protein was loaded onto a 12.5% (w/v) SDS-PAGE gel and transferred onto a nitrocellulose membrane. Antibodies against human p110*α* (Cell Signaling, Danvers, MA, USA, #4255), Akt (Cell Signaling, #9272), Bcl-2 (Cell Signaling, #2872) and epidermal growth factor receptor (EGFR; Cell Signaling, #2232) were used for estimating the amount of each protein. The antibodies against Phospho-Akt (Ser473; Cell Signaling, #9271), phosphor-Akt (Thr308; Cell Signaling, #9275) and phospho-EGFR (Tyr1045; Cell Signaling, #2237) at the indicated amino-acid residue sites were employed for determining phosphorylated protein level of Akt and EGFR, respectively.

### Flow cytometric analysis of F-actin contents

F-actin contents of HCT116 parental, WT and MUT cells were measured by Flow-Cytometry (BD LSRFortessa Cell Analyzer, BD Biosciences San Jose, CA, USA). In brief, cells were cultured in a dish to 90% confluence, trypsinized and subsenquently divided into 2-ml microtubes (2×10^6^ cells per tube), washed with PBS, fixed with 2% PFA for 1 h at room temperature, permeabilized with 0.1% Triton X-100 for 15 min and stained with Alexa Flour 488-conjugated phalloidin (A12379, Life Technologies) for 20 min at room temperature. After thorough washing with PBS containing 0.5% BSA, the cells were analyzed by flow cytometry and data analysis was carried out using Flow Jo (Tree Star Inc., Ashland, OR, USA).

### Wound-healing assay

Cell migration was studied using the scratch wound-healing assay. Four million HCT116 WT or MUT cells were plated into 6-well plates and incubated overnight. A sterile 200-*μ*l pipette tip was used to scratch three separate wounds. The cells were gently rinsed with DPBS and further incubated in 2.0 ml of culture medium. The wound closure was measured after 0, 6 or 12, and 24 h using a Nikon light microscope at ×10 magnification.

### PI3K inhibition by A66

The p110*α* specific inhibitor A66 (Selleck, Houston, TX, USA, #S2636) was used to inhibit p110*α*-PI3K activity. To test the effect of p110*α* inhibition on cell shape, HCT116 MUT cells were cultured on 18-mm-round coverslips for 18–24 h and subsequently treated with 1.0 *μ*M A66 for 0, 6, 12 and 24 h. Following the incubation with the inhibitor, cells were fixed and stained for F-actin. In order to investigate the effect of p110*α* inhibition on cellular Bcl-2 level, HCT116 WT and MUT cells were cultured in 100-mm dish for 24 h, after starved in serum-free medium overnight and before 20 min stimulation of 100 ng/ml of EGF, cells were treated for 3 h with 0, 0.1, 1.0 and 10.0 *μ*M of A66, respectively.

### Cell morphology analysis

Quantitative analysis of the cell morphology was based on the method of Analysis of Cell Morphology (http://amrita.vlab.co.in/?sub=3&brch=278&sim=1465&cnt=1). The area, perimeter and circularity of individual cells were measured using Image J (http://rsb.info.nih.gov/ij/). Cell morphology can be measured as a shape factor (*S*).

The shape factor (*S*) is determined using the following equation:

$$S=\frac{4\pi A}{P^{2}}$$

where *A*=the area of the cell and *P*=the cell perimeter.

Shape factor values range from 0 to 1, and measures the circularity or spreading of cells. Circular cells have a shape factor closer to 1, whereas a shape factor closer to 0 is indicative of an elongated cell.

### Statistical analysis

Statistical significance was determined using a two-tailed Student’s *t*-test with a *P-*value <0.05.

## Abbreviations

PI3K: phosphoinositide 3-kinase
PIP_3_: phosphatidylinositol (3,4,5)-trisphosphate (PtdIns(3,4,5)*P*_3_)
Bcl-2: B-cell lymphoma 2
CRC: colorectal cancer
WT: wild type
MUT: mutant
Akt: protein kinase B (PKB) or serine/threonine-specific protein kinase
EGFR: epidermal growth factor receptor
GFP: green fluorescent protein
