# Supplementary material for: The H1047R point mutation in p110 alpha changes the morphology of human colon HCT116 cancer cells
Source: Cell Death Discov. 2015 Oct 19;1:15044–. doi: 10.1038/cddiscovery.2015.44 (PMC4979441; doi:10.1038/cddiscovery.2015.44)
Supplement: Supplementary Figures [file cddiscovery201544-s1.pdf]

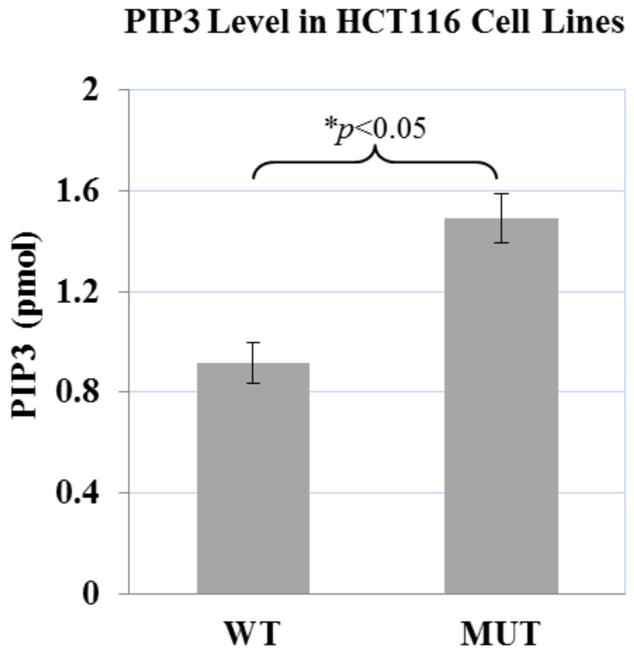

Supplemental Figure 1 **The H1047R mutation in the p110α kinase domain of PI3K increases the levels of cellular PIP_3.** Lipids were extracted from HCT116 WT and MUT cells (2.5×10^7 cells of each), and the levels of PIP_3 were measured using a PIP_3 Mass ELISA Kit. The level of PIP_3 was significantly higher in MUT cells than that in WT cells (two-tail student *t*-test, $p<0.05$).

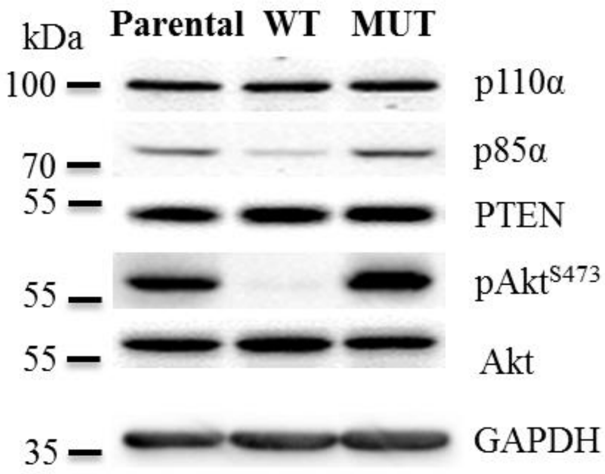

Supplemental Figure 2 **The H1047R mutation in the p110α kinase domain of PI3K increases phosphorylation of Akt.** p110α, p85α, PTEN and Akt levels in HCT116 cell lines were compared by Immuno-blotting analysis. HCT116 MUT cells show higher phosphorylated Akt levels compared to WT cells.
