# Supplementary material for: The H1047R point mutation in p110 alpha changes the morphology of human colon HCT116 cancer cells
Source: Cell Death Discov. 2015 Oct 19;1:15044–. doi: 10.1038/cddiscovery.2015.44 (PMC4979441; doi:10.1038/cddiscovery.2015.44)
Supplement: Supplementary Information [file cddiscovery201544-s2.pdf]

Supplemental Movies Movement of live HCT116 WT and MUT cells. HCT116 WT (Movie-1) and MUT (Movie-2) Cells, transfected with Lifeact-GFP, were cultured in a chamber slide for 24 hours, and transmission and fluorescence time-laps images were acquired over 10 minutes (40 × magnification).
